# Transplant-associated thrombotic microangiopathy in pediatrics: incidence, risk factors, therapeutic options, and outcome based on data from a single center

**DOI:** 10.3389/fonc.2024.1399696

**Published:** 2024-07-10

**Authors:** Kinan Kafa, Jessica I. Hoell

**Affiliations:** Department of Pediatric Hematology and Oncology, University Hospital Halle (Saale), Halle, Germany

**Keywords:** transplant-associated thrombotic microangiopathy, hematopoietic stem cell transplantation, eculizumab, multiorgan dysfunction syndrome, high-risk transplant-associated thrombotic microangiopathy, calcineurin inhibitor

## Abstract

**Background:**

Transplant-associated thrombotic microangiopathy (TA-TMA) is a critical complication of hematopoietic stem cell transplantation. Awareness about TA-TMA has increased in recent years, resulting in the implementation of TA-TMA screening in most centers.

**Methods:**

Retrospective analysis of children who underwent autologous or allogeneic hematopoietic stem cell transplantation at our center between January 2018 and December 2022 was conducted to evaluate the incidence, clinical features, and outcomes of TA-TMA following the administration of different therapeutic options.

**Results:**

A total of 45 patients comprised the study cohort, of whom 10 developed TA-TMA with a cumulative incidence of 22% by 100 days after transplantation. Patients with and without TA-TMA in our cohort displayed an overall survival of 80% and 88%, respectively (*p* = 0.48), and a non-relapse mortality of 0% and 5.7%, respectively (*p* = 0.12), at 1 year after transplantation. Risk factors for TA-TMA development included allogeneic transplantation and total body irradiation-based conditioning regime. Among the 10 patients with TA-TMA, 7 did not meet the high-risk criteria described by Jodele and colleagues. Of these seven patients, two responded to calcineurin-inhibitor withdrawal without further therapy and five developed multiorgan dysfunction syndrome and were treated with anti-inflammatory steroids (prednisone), and all responded to therapy. The three patients with high-risk TA-TMA were treated with complement blockade or prednisone, and all responded to therapy.

**Conclusion:**

TA-TMA is a multifactorial complication with high morbidity rates. Patients with high-risk TA-TMA may benefit from complement blockade using eculizumab. No consensus has been reached regarding therapy for patients who do not meet high-risk criteria. Our analysis showed that these patients may respond to anti-inflammatory treatment with prednisone.

## Introduction

Transplant-associated thrombotic microangiopathy (TA-TMA) is a serious complication of hematopoietic stem cell transplantation (HSCT) with high mortality and chronic organ injury (1). TA-TMA is a multifactorial complication characterized by endothelial dysfunction or injury, which leads to microangiopathic Coombs-negative hemolytic anemia, refractory thrombocytopenia, and multiorgan damage (2). Multiorgan damage may manifest as renal insufficiency, polyserositis, gastrointestinal bleeding, pulmonary hypertension, and/or encephalopathy (2–7).

TA-TMA can range from mild TA-TMA, which may need no therapy or only withdrawal of trigger medications [such as calcineurin inhibitors or mammalian target of rapamycin (mTOR) inhibitors], to severe TA-TMA, which has high mortality rates (7–9).

Histological confirmation remains the gold standard for TA-TMA diagnosis. The limited feasibility of invasive intervention after HSCT necessitates the development of non-invasive diagnostic criteria (1, 9–12). Most diagnostic criteria include elevated lactate dehydrogenase (LDH), low haptoglobin, thrombocytopenia, and the presence of schistocytes on peripheral blood smears (13).

The incidence of TA-TMA is unclear and varies from 0.5% to 76% according to the published literature (9). TA-TMA may develop after both allogeneic and autologous HSCT. Multiple risk factors mentioned in retrospective studies, including busulfan-based and total body irradiation (TBI)-based myeloablative conditioning, development of graft-versus-host disease (GvHD), GvHD prophylaxis using calcineurin inhibitors or mTOR inhibitors, and viral infection with cytomegalovirus, adenovirus, and human herpes virus 6, are associated with TA-TMA development (1, 8, 14–24).

The pathophysiology behind endothelial injury is still unclear. Recent studies have proposed a three-hit hypothesis: an underlying predisposition to compliment activation, toxic conditioning regimen, and post-transplant factors (14). Conditioning regimen, toxic medication, infections, and other transplant-related factors may induce endothelial injury, leading to increased protein inflammatory cytokine secretion that further promotes endothelial injury and activates the complement cascade (14).

Therapeutic options for treating TA-TMA with variable efficacy include plasma exchange, administration of rituximab, withdrawal of calcineurin inhibitor, and complement blockade (7, 9, 14, 20, 25–29). Newer published reports showed that patients have evidence of complement dysregulation (7, 30). This might suggest that complement blockade may be a therapeutic option for severe TA-TMA (7, 31–34). In recent studies, inhibition of complement activity has demonstrated promising results in selected patients with TA-TMA. The monoclonal antibody eculizumab binds C5 and inhibits terminal complement activation. Jodele and colleagues established a protocol to identify high-risk patients with TA-TMA (25). After administering eculizumab to the high-risk patients identified, 64% of the patients showed partial response (PR) and 56% achieved complete remission. Therefore, Jodele et al. recommend the early initiation of eculizumab and adjustment of its dosing to improve response.

In addition to classical complement pathway activation, the lectin complement pathway is activated by endothelial injury. Activation of lectin pathway also triggers the coagulation cascade, which also leads to the procoagulant phase, platelet adhesion, tissue injury, and finally to organ damage (35). Narsoplimab is a human monoclonal antibody that binds to mannan-binding lectin serine protease 2 (MASP2) and thereby blocks lectin-mediated complement activation without affecting the classical pathway. A phase II study administered narsoplimab in adults with severe TA-TMA and achieved high response rates (74%) in terms of laboratory markers and organ function without serious adverse effects (35, 36). Nevertheless, the lack of consensus on the standard treatment approach for TA-TMA makes it challenging to treat this complication.

In this paper, we describe our clinical experience involving a cohort of children who underwent transplantation at our center between January 2018 and December 2022.

## Methods

### TA-TMA screening

**Figure 1:**
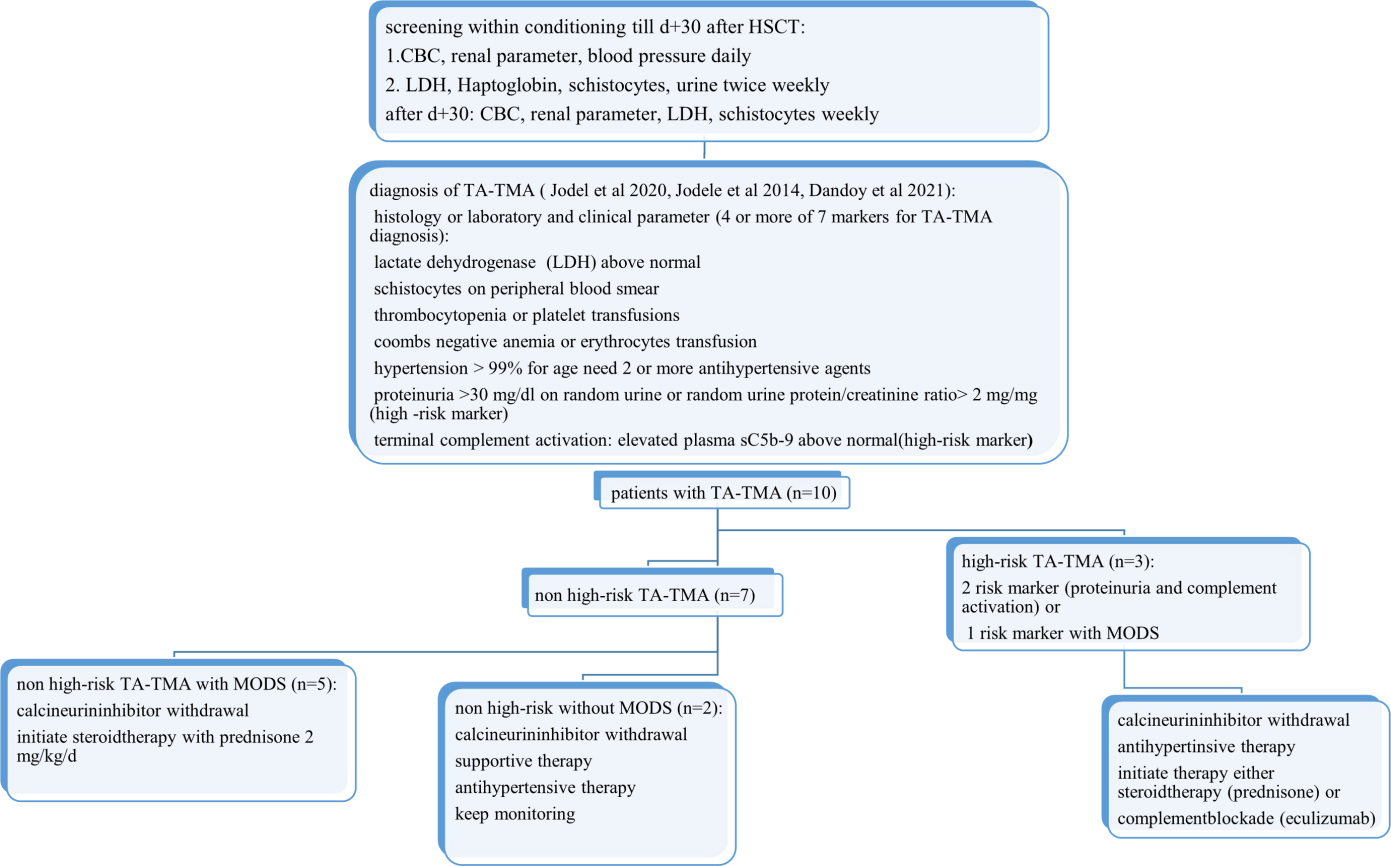
Screening and proposed therapy algorithm. LDH, lactate dehydrogenase; CBC, complete blood count. The patients were monitored for TA-TMA before conditioning them and until day 100 after transplantation or until TA-TMA resolves. Monitoring for TA-TMA in the first 30 days post-transplant included the evaluation of CBC, renal retention parameter (creatinine, urea, and cys-c GFR), blood pressure, LDH measured twice weekly, haptoglobin, schistocytes, and random urine protein/creatinine ratio in the urine specimen. The standard monitoring after day 30 post-transplant included weekly CBC, LDH, cys-c GFR, and schistocytes in blood smear. We applied the diagnostic criteria published earlier. TA-TMA was diagnosed either histologically based on a biopsy of affected organs or when four or more out of seven laboratory/clinical markers were simultaneously present and confirmed in two consecutive tests. These markers included elevated LDH, positive schistocytes, *de novo* thrombocytopenia, Coombs-negative hemolytic anemia, hypertension, and two high-risk criteria: proteinuria >30 mg/dL or random urine protein/creatinine ratio >2 mg/mg and terminal complement activation (c5b-9). Patients were classified as high risk if they fulfilled both high-risk criteria at diagnosis, or one high-risk criteria with evidence of multiorgan damage/dysfunction. All patients with TA-TMA received supportive therapy to heal the underlying organ injury, including antihypertensive therapy, minimized transfusion regimen, and management of acute infections and other transplant-related complications. GvHD prophylaxis with calcineurin or mTOR inhibitors were stopped for all patients within 2 weeks after diagnosis considering GvHD risk and time point post-HSCT with or without replacement therapy (mycophenolate-mofetil). High-risk patients with TA-TMA instantly received additional treatment with complement blockade (eculizumab) or high doses of prednisone 1–2 mg/kg/d tapered over 4–6 weeks. All patients with non-high-risk TA-TMA with multiorgan dysfunction syndrome (MODS) received anti-inflammatory therapy with prednisone 1–2 mg/kg/d followed by tapering over 4–6 weeks. Patients with non-high-risk TA-TMA without MODS were monitored closely without further therapy and received prednisone only if they did not respond to withdrawal of calcineurin inhibitors.

In our cohort, we utilize a screening and diagnosis algorithm according to evidence-based guidelines described in prior studies (1, 2, 9, 12, 30, 37–39) (**Figure 1**). The pediatric patients at our institution were monitored for TA-TMA before conditioning them and until day 100 after transplantation or until TA-TMA resolves. The routine monitoring for TA-TMA in the first 30 days post-transplant included daily blood count, renal function parameters [creatinine, urea, and cystatin c glomerular filtration rate (cys-c GFR)], blood pressure, LDH measured twice weekly, haptoglobin, the presence of schistocytes, and random urine protein/creatinine ratio in urine specimen. Complement activation (C50, C5b-9, and Anti H) was checked only if TA-TMA was suspected. The standard monitoring after day 30 post-transplant included weekly complete blood count, LDH, cys-c GFR, and the presence of schistocytes in blood smear. The laboratory characteristics are shown in **Table 1**.

**Table 1:** Laboratory characteristics and clinical risk factors in patients with and without TA-TMA.

	Patients with TA-TMA *N* = 10	Patients without TA-TMA *N* = 35	*p*-value
**Laboratory findings during the first 100 d after HSCT: LDH (U/L)**	507 (287–867)	281 (180–374)	*p* < 0.001
**No. of platelet transfusions in the first 100 d**	11 (4–32)	10 (1–32)	*p* = 0.071
**No. of erythrocyte transfusions in the first 100 d**	8 (3–15)	6 (1–13)	*p* = 0.087
**Days to platelet engraftment > 20 × 10^9^/L**	38 (12–150)	25 (11–120)	*p* = 0.2
**Days to platelet engraftment > 50 × 10^9^/L**	49 (12–170)	30 (12–150)	*p* = 0.098
**AKI (doubling of creatinine)**	8 (80%)	7 (20%)	*p* < 0.001
**Clinical risk factors**			
**aGvHD Grade II–IV**	2 (20%)	12 (34.2%)	*p* = 0.63
**Viral infection**	3 (30%)	11 (31.4%)	*p* = 0.74
**VOD/SOS**	0	4 (11.4%)	*p* = 0.26

TA-TMA, transplant-associated thrombotic microangiopathy; aGvHD, acute graft-versus-host disease; HSCT, hematopoietic stem cell transplantation; VOD/SOS, hepatic veno-occlusive disease (VOD) or sinusoidal obstruction syndrome (SOS); AKI, acute kidney injury; LDH, lactate dehydrogenase (reference 100–250 U/L).

### Definition of TA-TMA and risk stratification

We applied the diagnostic criteria published by Jodele et al. in 2014 and Dandoy et al. in 2020 (2, 12, 18, 25, 37, 39–41). TA-TMA was diagnosed either histologically based on biopsy of affected organs or when four or more laboratory/clinical markers out of seven markers were simultaneously present and confirmed in two consecutive tests. These markers included (1) elevated LDH, (2) presence of schistocytes in blood smear, (3) *de novo* thrombocytopenia (number of thrombocytes < 50 × 10^9^/L) or >50% decrease in platelet count, (4) Coombs-negative hemolytic anemia or increasing need for blood transfusion, and (5) hypertension > 99th percentile for age along with the high-risk criteria: (6) evidence of kidney injury with proteinuria (protein level in urine > 30 mg/dL) or random urine protein/creatinine ratio > 2 mg/mg and (7) terminal complement activation (C5b-9).

Patients were classified as high risk if they fulfilled both high-risk criteria at diagnosis or one high-risk criteria with evidence of multiorgan damage/dysfunction [multiorgan dysfunction syndrome (MODS)] (2, 14, 25, 41–49).

### Definition of multiorgan dysfunction syndrome

TA-TMA affects a wide spectrum of organs and may result in acute or chronic organ dysfunction. This includes renal injury, hypertension, intestinal bleeding, encephalopathy, pulmonary hypertension, and polyserositis (1, 2, 14, 25). Patients with TA-TMA with MODS are critically sick and need prompt therapeutic interventions although these patients do not always fulfill the high-risk criteria for TA-TMA.

### Patients and clinical data

All patients who underwent allogeneic and autologous HSCT at our institution between January 2018 and December 2022 were included in this cohort (*n* = 35 and *n* = 10, respectively). The indication for autologous HSCT was high-risk neuroblastoma in seven cases and relapsed Ewing sarcoma, germ-cell cancer, and relapsed nephroblastoma in one case each. Patient data were collected, including sex, transplant diagnosis, stem cell source, conditioning regimen (50), GvHD prophylaxis, transplantation-related complications, and outcome. GvHD was diagnosed according to the Glucksberg clinical criteria (51). The clinical characteristics are shown in **Table 2**. We divided the participants with TA-TMA in our cohort according to the high-risk criteria into three groups: (1) high-risk TA-TMA (2, 14, 25), (2) non-high-risk TA-TMA with MODS (1, 14, 25), and (3) non-high-risk TA-TMA without MODS.

**Table 2:** Baseline clinical characteristics of patients who underwent HSCT between January 2018 and December 2022.

	Patients with TA-TMA *N* = 10	Patients without TA-TMA *N* = 35	*p*-value
**Male sex**	5 (50%)	21 (60%)	0.46
**Age, years**	11 (2–17)	6 (1–17)	
**Age**			0.24
**>10 years**	4 (40%)	8 (22.8%)	
**<10 years**	6 (60%)	27 (77.2%)	
**Initial diagnosis**			0.46
** Malignancy**	5 (50%)	23 (65.7%)	
** Bone marrow failure (MDS, SAA)**	3 (30%)	5 (14%)	
**Immunodeficiency/metabolic disease**	1 (10%)	4 (11.4%)	
** Benign hematologic disease**	1 (10%)	3 (8.5%)	
**HSCT-Typ**			0.29
** Allogeneic**	9 (90%)	26 (74.3%)	
** MUD (9 or 10/10)**	8 (89%)	21 (80.7%)	
** MFD**	0	2 (7.7%)	
** MMFD (haploidentical)**	1 (11%)	3 (11.5%)	
** Autologous**	1 (10%)	9 (25.7%)	
**Stem cell source**			0.037
** BM**	4 (40%)	4 (11.4%)	
** PBSC**	7 (70%)	30 (85.7%)	
**Conditioning regimen**			0.098
** Bu-based regimen**	2 (20%)	10 (28.5%)	
** TBI-based regimen**	3 (30%)	2 (5.7%)	0.031
** Other**	5 (50%)	23 (65.7%)	
**GvHD prophylaxis**			0.75
** CSA-based prophylaxis**	8 (80%)	24 (68.6%)	
** MMF-based prophylaxis**	1 (10%)	2 (5.7%)	

TA-TMA, transplant-associated thrombotic microangiopathy; GvHD, graft-versus-host disease; HSCT, hematopoietic stem cell transplantation; MDS, myelodysplastic syndrome; SAA, severe aplastic anemia; BM, bone marrow; pBSC, peripheral blood stem cell; CSA, ciclosporin A; Bu, busulfan; TBI, total body irradiation; MMF, mycophenolate-mofetil.

The study was approved by the local ethics committee of our institution.

### Statistical analysis

SPSS statistical software, version 29 (IBM^®^, New York, USA) was used to analyze the collected data. Pearson’s chi-squared test was used to determine any association between age, gender, cell source, HSCT type, conditioning, etc. as risk factors and TA-TMA development. Moreover, *t*-test and one-factor analysis of variance were used to test whether the average values of multiple clinical and laboratory factors between the patients with and without TA-TMA and among the TA-TMA groups are different. The results are expressed as an odds ratio (OR) with a 95% confidence interval (CI), and data were considered significant at *p*-value < 0.05.

## Results

### Cohort characteristics

We identified 45 pediatric patients who underwent allogeneic or autologous HSCT at our institution from January 2018 to December 2022. The clinical characteristics are shown in **Table 2**. Malignant diseases were the most common underlying diagnosis (*n* = 28, 62%). Moreover, 35 subjects received allogeneic transplants (77.8%) from 9/10 or 10/10 matched unrelated donor (*n* = 30, 85.8%), matched family donor (*n* = 2, 5.7%), or haploidentical donor (*n* = 3, 8.5%), and the most frequent cell source was peripheral blood stem cells (*n* = 37, 82%). Nearly all transplant recipients (95.5%) received TBI-, busulfan-, or treosulfan-based myeloablative conditioning. Ciclosporin A was the most frequently used GvHD prophylaxis (*n* = 32, 91.4%).

Ten of the 45 patients (22%) met the criteria for TA-TMA diagnosis. High-risk TA-TMA was diagnosed in 6.6% of all HSCT patients (*n* = 3), of which two cases occurred after allogeneic HSCT and one case occurred after autologous HSCT with high-risk neuroblastoma.

All affected patients developed this complication within the first 100 days after transplantation with a median of 49 (11–98) days. Patients with high-risk TA-TMA were more likely to be older in comparison to non-high-risk patients [median age, 14 (8–17) years vs. 8.5 (2–17) years, respectively] and developed pericardial/pleural effusions more frequently (*p* = 0.01). No patient in our cohort had evidence of complement activation (C5b-9 range: 98–191 ng/mL). Patients with or without high-risk TA-TMA showed no difference in conditioning regimen (*p* = 0.098), GvHD rates (30% vs. 31%, *p* = 0.63), and documented infection (20% vs. 34.2%, *p* = 0.74). The demographics and characteristics of patients with TA-TMA are shown in **Table 3**.

**Table 3:** Demographics and disease characteristics of patients with TA-TMA.

	High-risk TA-TMA			Non-high-risk TA-TMA						
Patients	No. 1	No. 2	No. 3	No. 4	No. 5	No. 6	No. 7	No. 8	No. 9	No. 10
**Age at SCT, years**	<10	>10	>10	<10	<10	>10	<10	<10	<10	>10
**Diagnosis**	M	B	M	M	M	B	B	B	M	B
**Stem cell source**	pB	pB	pB	BM	BM	BM	pB	pB	BM	pB
**Conditioning regimen**	TBI/VP16	Treo/Fl/TT	Bu/Mel	TBI/VP16	TBI/VP16	Fl/Cy	Bu/Fl/Mel	Treo/Fl	Treo/Fl/TT	Treo/Fl
**Days after SCT at diagnosis**	92	43	16	21	100	11	47	17	54	98
**Cys c-GFR mL/min/1.73 m²**	49	62	83	50	91	59	108	66	90	42
**Proteinuria (protein level in urine > 30 mg/dL) or protein-creatinine ratio > 2 mg/mg**	31 mg/dL	26 mg/dL, P/C ratio: 3.3 mg/mg	30 mg/dL	No	No	6 mg/dL	14 mg/dL	5 mg/dL	11 mg/dL	26 mg/dL
**Transplant-related complication**	Serositis	Serositis	Serositis	Serositis, encephalopathy	Serositis	Serositis	PHT	Intestinal bleeding, serositis	No	No
**Complement C5b-9 (ng/mL)**	150	125	191	121	170	156	107	133	98	188
**Biopsy**	Kidney	ND	ND	ND	ND	ND	ND	Colon	ND	ND
**GvHD stage, organ**	Grade I, skin	Grade I, skin	NA	Grade II, skin	No	Grade II, skin	No	No	No	No
**Infections**	No	Herpes simplex	No	EBV/CMV	No	EBV	No	No	No	No
**Therapy**	Eculizumab CNI withdrawal	Eculizumab+ Pred + CNI withdrawal	Pred	CNI withdrawal + Pred	CNI withdrawal + Pred	CNI withdrawal + Pred	CNI withdrawal + Pred	CNI withdrawal + Pred	CNI withdrawal	CNI withdrawal
**Response**	CR	PR	CR	CR	PR	CR	CR	CR	CR	CR

M, malignant; B, benign; NA, not applicable; ND, not done; CR, complete response; PR, partial response; BM, bone marrow; pB, peripheral blood stem cells; CNI, calcineurin inhibitor; TBI, total body irradiation; VP16, etoposide; Bu, busulfan; Treo, treosulfan; Flu, fludarabine; Mel, melphalan; Cy, cyclophosphamide; Pred, prednisone; GvHD, graft-versus-host disease; complement C5b-9, reference 58–239 ng/mL; PHT, pulmonary hypertension; P/C ratio, protein/creatinine ratio in spontaneous urine sample.

### Clinical monitoring

The number of erythrocyte and platelet transfusions was documented for 100 days after transplantation. Red blood transfusion was administered at a hemoglobin level < 7 g/dL and platelet count < 20 × 10^9^/L. Hypertension was defined when systolic and/or diastolic values > 99th percentile for age. The number of antihypertensive medications for each patient was also documented. Cytomegalovirus, adenovirus, and Epstein–Barr virus screening via polymerase chain reaction were performed at least once weekly.

A diagnosis of acute kidney injury was reached if the creatinine values were doubled compared to the baseline value before transplantation (2, 40, 52).

The occurrences of MODS, GvHD, and transplantation-related complications were noted for the first 100 days after transplantation.

### Risk factors for TA-TMA

Among the 45 subjects, TA-TMA was more likely to develop after allogeneic HSCT than after autologous HSCT (25.7% vs. 10%, respectively, *p* = 0.29). All TA-TMA cases that developed after allogeneic HSCT were seen in patients who received transplants from matched unrelated donors. No statistical difference in TA-TMA rates was seen between allogeneic transplantations from 9/10 or 10/10 unrelated donors (40% vs. 24%, *p* = 0.4). The risk was slightly higher in patients with non-malignant disease in comparison to those with malignant diseases in the whole cohort (29.4% vs. 17.8%, *p* = 0.46) and in the allogeneic HSCT (29.4% vs. 22.2%, *p* = 0.46) both without statistical significance. As a cell source, only bone marrow showed a significant difference (50% vs. 18.9%, *p* = 0.037), although the results should be considered carefully considering the low patient numbers in our cohort.

Notably, the risk of developing TA-TMA was higher in older patients (>10 years old), but without statistical significance. Out of 12 patients in our cohort who were older than 10 years, 4 developed TA-TMA, compared to 6 out of 33 who were younger (33.3% vs. 18%, *p* = 0.24). GvHD prophylaxis with ciclosporin A was not an independent risk factor for TA-TMA (33.3% vs. 25%, *p* = 0.75), given that it was used in all cases and there was no comparison group.

Nearly all subjects received myeloablative conditioning (95.5%). We observed that patients who received TBI-based conditioning were more likely to develop TA-TMA (60% vs. 17.5%, *p* = 0.031). Busulfan- and treosulfan-based regimens did not appear to be a significant risk factor for TA-TMA development (16.6% vs. 24.2, *p* = 0.7 and 26% vs. 18.2%, *p* = 0.72, respectively).

Severe acute GvHD (Grades III and IV) was not a significant risk factor for TA-TMA development (30% vs. 31%, *p* = 0.63), and infections in the first 100 days after transplantation were similarly not associated with an increased risk for TA-TMA. Our cohort of patients who developed other types of angiopathy complications (hepatic veno-occlusive disease or sinusoidal obstruction syndrome) do not exhibit a significantly increased risk of developing TA-TMA (0% vs. 11.4%, *p* = 0.26).

### Treatment of TA-TMA

Recent studies have tried to develop treatment strategies based on TA-TMA pathophysiology (2, 14, 25, 53). Several have reported that calcineurin inhibitors such as ciclosporin and tacrolimus and mTOR inhibitors such as sirolimus might be probable causal factors for endothelial injury in patients with TA-TMA (1, 8, 9, 23, 54, 55).

All patients with TA-TMA at our institution received supportive therapy to heal the underlying organ injury, which included antihypertensive therapy, minimized transfusion regimen (2, 14, 25, 39), and management of acute infections and other transplant-related complications.

GvHD prophylaxis with calcineurin or mTOR inhibitors were stopped in all patients within 2 weeks after diagnosis considering GvHD risk and the time point after administering HSCT with or without replacement therapy (mycophenolate-mofetil) (8, 9, 23, 54, 56, 57). Patients with high-risk TA-TMA instantly received additional treatment with complement blockade (eculizumab) (7, 14, 18, 25, 27–29, 58, 59) or high doses of prednisone 1–2 mg/kg/d and tapering over 4–6 weeks.

All patients with non-high-risk TA-TMA with MODS received anti-inflammatory therapy with prednisone 1–2 mg/kg/d followed by tapering over 4–6 weeks (14). Patients with non-high-risk TA-TMA without MODS were monitored closely without further therapy and received prednisone only if they did not respond to calcineurin inhibitor withdrawal.

### Classification of treatment response

Response to therapy was evaluated once a week during the therapeutic regimen and after it ended. Overall survival was evaluated at 3, 6, and 12 months after transplantation. Patients were defined as displaying complete response (CR) if MODS, transfusion-dependent anemia, and thrombocytopenia resolved completely (25). Patients with no response (NR) still have active disease with MODS or still need transfusion of erythrocytes and/or thrombocytes. All other patients, who did not meet NR or CR criteria or who had relapsed after therapy stopped, were defined as having PR.

### Complications of TA-TMA and therapeutic response

Our cohort showed variability in the clinical presentation and disease management of TA-TMA. Patient demographics and disease characteristics are shown in **Table 3**.

Renal impairment and chronic kidney disease were the most frequent complications in patients with TA-TMA (*n* = 8, 80%), followed by (poly)serositis that resulted in pleural and pericardial effusion and ascites (*n* = 7, 70%). Pulmonary hypertension (*n* = 1, 10%), gastrointestinal bleeding (*n* = 1, 10%), and encephalopathy (*n* = 1, 10%) were also documented. No patient in our study showed complement activation at TA-TMA diagnosis. The cys-c GFR of most patients with significant renal injury were still below the pretransplantation cys-c GFR after the patients recovered from TA-TMA (*n* = 6, 60%) and needed long-term follow up.

Cessation of immunosuppression by withdrawing calcineurin inhibitors or switching to mycophenolate-mofetil was the first therapeutic strategy employed in all patients with TA-TMA.

Patients with TA-TMA also received adjunct therapy such as complement blockade with eculizumab or anti-inflammatory agents with prednisone according to high-risk stratification or existing critical organ damage/dysfunction as seen in MODS. Patients with high-risk TA-TMA (*n* = 3, 30%) were treated with eculizumab (*n* = 1, 10%) and prednisone (*n* = 2, 20%). Two patients achieved CR (66%), while one patient showed PR (33%) to therapy with prednisone and was switched to eculizumab. The number of eculizumab doses was 11, and the duration of therapy was 113 days. Therapy was well tolerated without any significant side effects. Response to therapy is described in **Table 4**.

**Table 4:** Response to therapy.

	High-risk TA-TMA n=3	Non-high-risk TMA MODS+ n=5	Non-high-risk TMA MODS− n=2	*p*-value
**Age, years**	14 (8–17)	7.8 (2–17)	10.5 (4–17)	*p* = 0.43
**Time until normalization of LDH, d**	23.6 (7–44)	27.8 (11–74)	94 (48–140)	*p* = 0.095
**Time until normalization of haptoglobin, d**	98 (14–225)	99.6 (78–230)	115 (80–150)	*p* = 0.922
**Time until disappearance of schistocytes, d**	45 (12–75)	19.6 (14–30)	96.5 (43–150)	*p* = 0.078
**Time until platelets were recovered to >20 × 10^9^/L**	25.3 (12–40)	35.2 (12–80)	37.5 (30–45)	*p* = 0.8
**Time until TA-TMA resolution, d**	65.8 (36–105)	65.8 (26–120)	105 (60–150)	*p* = 0.54

TA-TMA, transplant-associated thrombotic microangiopathy; MODS, multiorgan dysfunction syndrome; MODS+, with MODS; MODS−, without MODS; LDH, lactate dehydrogenase; d, days; Y, years.

Patients who did not meet the high-risk criteria were divided into critically sick patients with MODS who received therapy with prednisone (*n* = 5, 50%) and patients without MODS who did not receive adjunct treatment (*n* = 2, 20%). All patients with non-high-risk TA-TMA with MODS showed CR to prednisone therapy with a median treatment duration of 38 (30–48) days (**Figure 1**).

It is worth mentioning that withdrawal of calcineurin inhibitors was initiated in many patients concurrently with the initiation of corticosteroid administration (6/10 patients with TA-TMA, 60%, 1 with high-risk TA-TMA and 5 with non-high-risk TA-TMA) (**Table 3**). Therefore, it is not clear whether the withdrawal of calcineurin inhibitors by itself possibly resolved TA-TMA without the addition of steroids.

TA-TMA in all patients without MODS (*n* = 2, 20%) resolved spontaneously approximately 14 (12–21) days after ceasing immunosuppression without adjunct treatment.

The adjusted analysis showed that hematologic resolution occurred in 77 (36–105) days and 77 (26–150) days in high-risk and non-high-risk TA-TMA, respectively.

**Figure 2:**
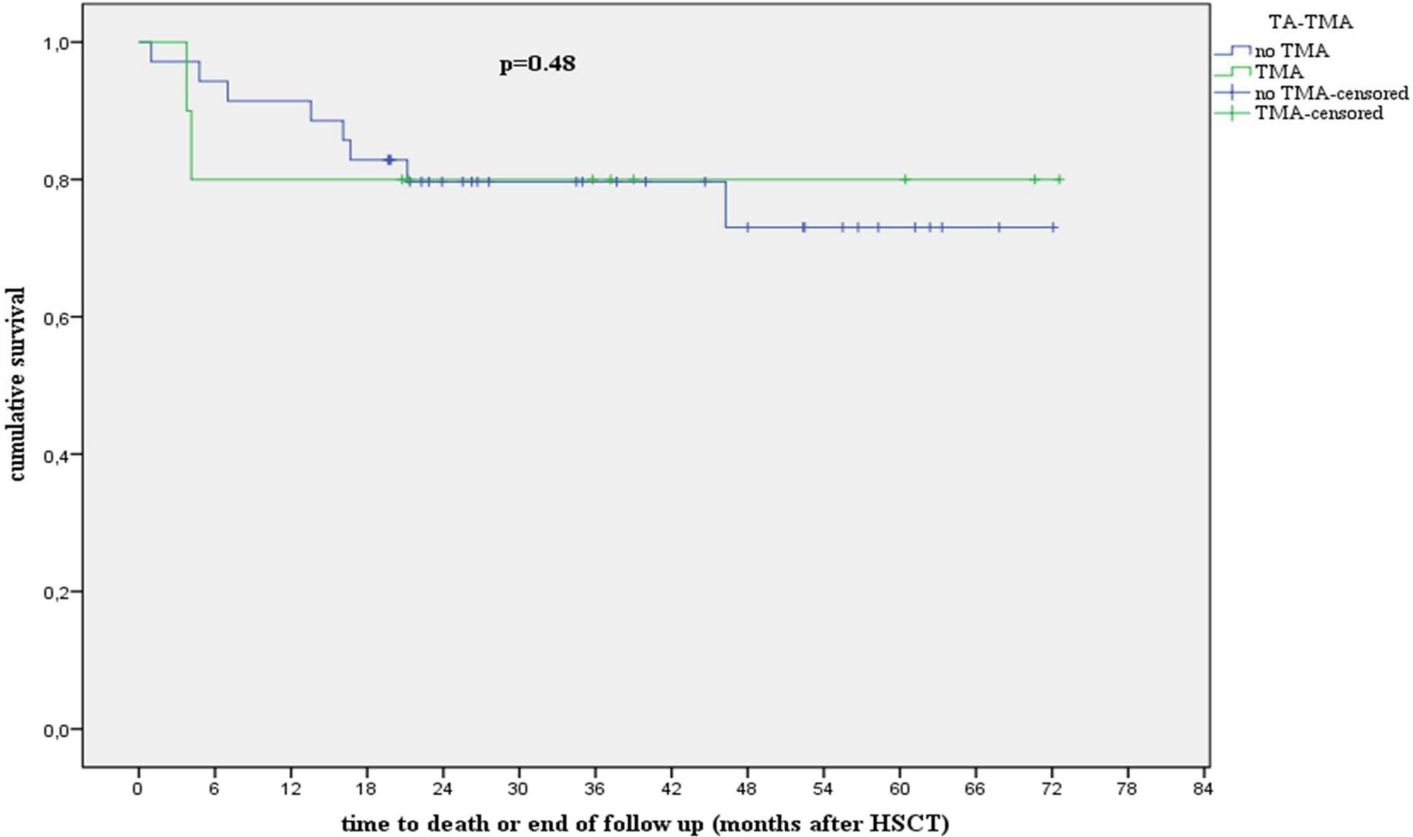
Analysis showing survival in patients with and without TA-TMA. hR, high risk; HSCT, hematopoietic stem cell transplantation; TA-TMA, transplant-associated thrombotic microangiopathy. We compared the overall survival (OS) and non-relapse mortality (NRM) 1 year after stem cell transplantation. Patients with and without TA-TMA in our cohort exhibited an OS (*p* = 0.48) of 80% and 88%, respectively, and an NRM (*p* = 0.12) of 0% and 5.7%, respectively, at 1 year after transplantation.

Eight of 10 patients with TA-TMA in our cohort were alive 1 year after diagnosis with an overall survival of 80% and non-relapse mortality of 0% (**Figure 2**).

## Discussion

In this paper, we report our experience in the diagnosis and management of patients with newly diagnosed TA-TMA after HSCT. The cohort had 45 pediatric transplant recipients who underwent allogeneic or autologous HSCT. The overall incidence of TA-TMA reported in the literature varies widely from 0.5% to 76%. This wide range may be attributed to the fact that most recent studies are retrospective observations; moreover, mild TA-TMA cases may have remained undiscovered (2, 12, 17, 60).

In our cohort, TA-TMA was documented in 22% (*n* = 10) of all patients based on currently published diagnostic criteria. All cases occurred within 100 days after transplantation with a median time of 49 (11–98) days.

Several studies have observed a high risk of TA-TMA following autologous HSCT in children (2, 7, 61). In our study, only 1 patient out of 10 autologous HSCTs with high-risk neuroblastoma developed TA-TMA, giving rise to a TA-TMA incidence of 10%. A single-centered study by Schoettler et al. described pediatric TA-TMA following autologous HSCT. The incidence of TA-TMA was 3.7%, which occurred most frequently in patients with neuroblastoma (78%), all of whom were conditioned with carboplatin, etoposide, and melphalan. Consistent with our observations, TA-TMA was diagnosed within the first 100 days after transplantation. Most of these patients had normal levels of complement and had renal involvement at presentation. In the study by Schoettler et al., the prevalence of TA-TMA in patients with neuroblastoma was 6%, which is low compared to 14% (1 of 7) in our study and significantly lower than that mentioned in the literature (30%). Considering that all the patients with neuroblastoma in our cohort were conditioned with busulfan and melphalan, we believe that patients with neuroblastoma may have additional risk factors related to the disease itself. These factors may include endothelial injury caused by the initially high levels of catecholamine in combination with standard chemotherapy apart from conditioning regimen (62).

When examining the risk factors associated with TA-TMA, we found a higher incidence of TA-TMA among patients who received a conditioning regimen with TBI (60 vs. 17.5%, *p* = 0.031), were older than 10 years (33.3 vs. 18%, *p* = 0.24), and had non-malignant diseases (29.4% vs. 17.8%, *p* = 0.46) (2); however, GvHD prophylaxis with calcineurin inhibitors did not seem to be an independent risk factor for TA-TMA development (33.3% vs. 25%, *p* = 0.75). Similar results were reported by Higham et al. in 2021, who described a retrospective analysis of TA-TMA with 257 pediatric patients who underwent 292 allogeneic HSCTs (63). They showed higher incidence of TA-TMA in patients aged >10 years than in those aged <10 years (9.8% vs. 3.1%, *p* = 0.4) and in patients who received TBI-based myeloablative conditioning regimens compared with non-TBI-based regimens (12.2% vs. 5.7%, *p* = 0.17) with no impact from the stem cell source.

Contrary to our results, severe aplastic anemia as an underlying disease was found to be a clear risk factor compared with patients with malignancy or other non-malignant diagnoses (17.6% vs. 8.3% vs. 0%, *p* = 0.006). However, we detected higher rates of TA-TMA in patients with bone marrow failure, including severe aplastic anemia and myelodysplastic syndrome, who underwent allogeneic HSCT compared to malignant or other non-malignant diseases (37.5% vs. 22.2% vs. 22.2%, *p* = 0.68) (63).

Patients who met the criteria of TA-TMA have a high risk of post-transplant morbidity and may develop multiple clinically significant complications including pulmonary hypertension, severe gastrointestinal bleeding, and pleural and pericardial effusion. This supports the observations of previous studies that TA-TMA may lead to multiorgan damage/dysfunction, which coexist with other HSCT complications (2, 14, 64–69).

The TA-TMA severity in our cohort ranged from mild, self-limiting TA-TMA to severe, high-risk TA-TMA with proteinuria, renal injury, multiorgan damage, and high risk of morbidity and admission to intensive care unit (2). Mild cases resolved with supportive care only, including antihypertensive therapy, minimization of transfusions, and withdrawal of calcineurin inhibitors (9, 14). High-risk TA-TMA has successfully been treated with different medications. Based on recent studies on the efficacy and safety of complement blockade in patients with high-risk TA-TMA, one patient was treated with eculizumab while the other two patients were treated with prednisone in the present study (9, 14, 25, 39, 53, 70, 71). TA-TMA was resolved in one of the two patients who received prednisone; however, prednisone therapy had to be switched to eculizumab therapy in the other patient due to relapse after initial response. This patient showed PR to eculizumab and developed chronic renal insufficiency. It remains unknown why the two patients showed a clinical response with complement blockade despite no evidence of complement activation being detected in the laboratory.

In our cohort, we observed a group of patients with non-high-risk TA-TMA (*n* = 5, 50% of all patients with TA-TMA), who were critically sick with MODS and TA-TMA-related complications. To our knowledge, no consensus exists regarding therapy for these types of patients.

Recent publications examining TA-TMA pathophysiology, which hypothesized that endothelial injury leads to an increase in proinflammatory cytokine, procoagulant factors, and adhesion molecules, led us to consider interrupting the inflammatory cascade with prednisone. Since the stimulation of endothelial injury in most cases is temporary, we tapered prednisone over 4–6 weeks (14, 25).

All five patients responded to therapy with hematological and MODS resolution in 65 (26–120) days. Most patients with TA-TMA with significant renal injury still exhibited a cys-c GFR that was below the pretransplantation value after TA-TMA recovery and required long-term follow up.

Here, we describe our experience with the risk factors, outcome, and impact of TA-TMA treatment on subsequent transplant outcomes.

We recognize several limitations of our analysis. First, the study represents a small number of patients with TA-TMA with a relatively small total sample size, making it difficult to interpret the results and their significance and to draw definite conclusions. Second, the retrospective design of the cohort may lead to underreporting less severe TA-TMA cases. Furthermore, opportunities for histological confirmation of the TA-TMA diagnosis in this vulnerable population of patients after HSCT with high bleeding risk were limited.

Despite limitations, this retrospective study provides crucial data showing that TA-TMA impairs the quality of life of a significant proportion of pediatric transplant recipients. Additionally, patients undergoing HSCT should have a routine scheduled screening program to identify suspected cases early to prevent lasting organ damage.

Multicenter prospective studies will be needed to construct a consensus recommendation for early recognition and monitoring of children with this common post-transplant complication and for developing a therapeutic approach tailored according to risk.

## Data availability statement

The original contributions presented in the study are included in the article/supplementary material. Further inquiries can be directed to the corresponding author.

## Ethics statement

The studies involving humans were approved by Ethics Committee, University Halle (Saale). The studies were conducted in accordance with the local legislation and institutional requirements. The ethics committee/institutional review board waived the requirement of written informed consent for participation from the participants or the participants’ legal guardians/next of kin because purely retrospective analysis performed.

## Author contributions

KK: Writing – original draft, Writing – review & editing. JH: Writing – original draft, Writing – review & editing.
